# SIRT1 increases cardiomyocyte binucleation in the heart development

**DOI:** 10.18632/oncotarget.23847

**Published:** 2018-01-03

**Authors:** Alexandra N. Shin, Limin Han, Chiranjib Dasgupta, Lei Huang, Shumei Yang, Lubo Zhang

**Affiliations:** ^1^ The Lawrence D. Longo MD Center for Perinatal Biology, Department of Basic Sciences, Loma Linda University School of Medicine, Loma Linda, California, USA; ^2^ Department of Biological Sciences, California Baptist University, Riverside, California, USA; ^3^ Department of Chemistry and Biochemistry, California State University, San Bernardino, California, USA

**Keywords:** binucleation, hypoxia, miR-133a, SIRT1, cardiomyocyte

## Abstract

SIRT1 regulates cell senescence. We investigated a novel role of SIRT1 in the regulation of cardiomyocyte terminal differentiation in the developing heart. Retinoic acid (RA)-induced binucleation of H9c2 cells was associated with increased SIRT1 expression. Inhibition of SIRT1 activity or expression significantly decreased RA-induced binucleation. SIRT1 expression was minimal in the fetal heart and significantly upregulated in the hearts of postnatal day 7 (P7) rat pups. In contrast, heart-specific miR-133a expression was high in the fetal heart but significantly reduced in P7 pup hearts. The miR-133a promoter contains a canonical HRE element and hypoxia upregulated miR-133a gene expression in the heart. SIRT1 mRNA 3′UTR has miR-133a binding sequences and miR-133a and hypoxia suppressed SIRT1 expression in cardiomyocytes. Of importance, inhibition of SIRT1 significantly reduced binucleated cardiomyocytes in the hearts of P7 pups. Taken together, the present study reveals a novel role of SIRT1 and its regulation by miR-133a in cardiomyocyte terminal differentiation of the developing heart, and suggests a potential therapeutic strategy that may impact cardiac function later in life.

## INTRODUCTION

Cardiomyocytes are the functional units of the heart, and undergo terminal differentiation as they mature from fetal to adult hearts [1–4]. This terminal differentiation process occurs around birth, in which the cardiomyocytes exit the cell cycle, become binucleated and lose their ability to proliferate [1, 5–7]. Adult cardiomyocytes have lost their proliferative capacity and thus have negligible increases in number following birth [8]. Therefore, the timing of cardiomyocytes in losing proliferation may ultimately determine the number of cardiomyocytes endowed in the heart for life. However, the mechanisms and factors that control this transition remain largely unknown.

The silent information regulator of transcription (SIRT) family of proteins include a group of class III lysine deacetylases that regulate various intracellular processes including metabolism [9, 10], oxidative stress [11–13], and apoptosis [11, 12]. Of particular interest is its role in regulating the cell cycle [12, 14–16] and senescence [13, 14]. Activation of SIRT1 is dependent on NAD^+^ [17–20] and is directly tied to the energy and redox status of the cell [9, 21–23]. SIRT1 can also be activated through nicotinamide-depletion, whereas nicotinamide (NAM) itself is shown to inhibit SIRT1 activity [24].

In the present study, we sought to investigate a possible role of SIRT1 in the regulation of cardiomyocyte terminal differentiation in the developing heart. Herein, we present a novel finding that SIRT1 promoted cardiomyocyte binucleation, and inhibition of SIRT1 in newborn rat pups impeded cardiomyocyte maturation at the critical window of heart development after birth to postnatal day 7 rat pups. We demonstrated that SIRT1 expression was extremely low in the fetal heart and was significantly upregulated after birth. Fetal development takes place in a hypoxic environment, in which hypoxia inducible factor (HIF) is a key transcription factor that regulates various aspects of embryonic development [25]. We identified that microRNA-133a (miR-133a), a miR specifically expressed in cardiac muscle [26, 27] and involved in cardiomyocyte proliferation [28, 29], was upregulated by hypoxia due to a classical hypoxia response element (HRE) present in the promoter of miR-133a. Of importance, the present study revealed that SIRT1 was a downstream target of miR-133a and the 3′UTR of SIRT1 transcript harbored a miR-133a recognition sequence. Thus, these findings implicate miR-133a/SIRT1 as key regulators in cardiomyocyte terminal differentiation and maturation during the heart development.

## RESULTS

### Inhibition of SIRT1 decreased RA-induced binucleation of H9c2 cells

**Figure 1:**
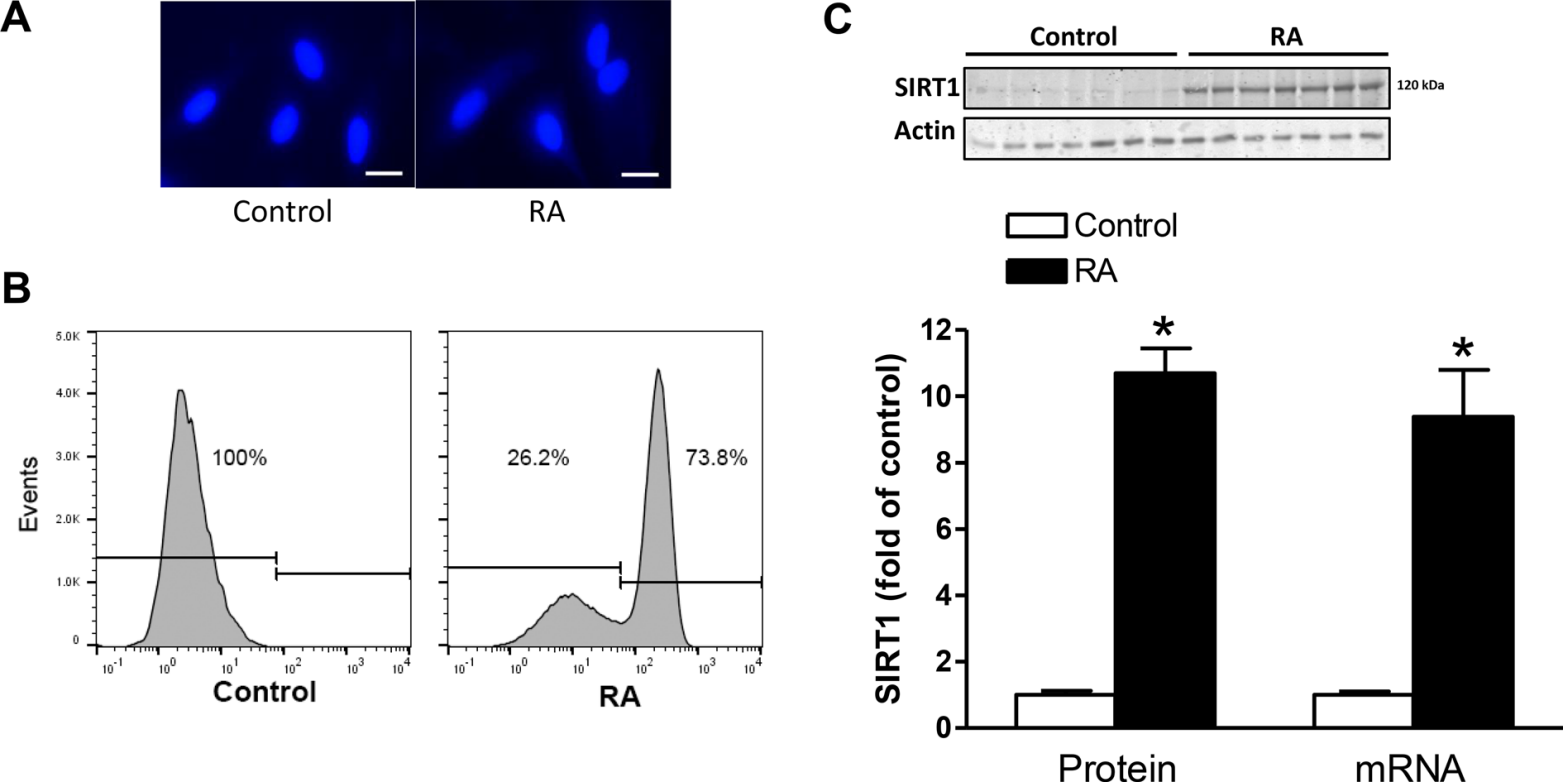
RA increased SIRT1 expression and induced binucleation of H9c2 cells H9c2 cells were treated with 1 μM retinoic acid (RA) for 5 days to induce binucleation. (**A**) Nuclei labeled with Hoechst (blue). Scale bar = 100 μm. (**B**) The percentage of mononucleated and binucleated cells in the absence (control) or presence of RA. (**C**) SIRT1 protein and mRNA abundance in the absence (control) or presence of RA. Data are the means ± SEM. *n* = 6–7 ^*^P < 0.05, RA *vs.* control.

The H9c2 cell line was developed from the embryonic rat heart [30]. When cultured in 10%-serum medium, H9c2 cells grow as mononucleated cells, as identified by DNA specific staining with Hoechst reagent (Figure 1A, left panel). Treatment of H9c2 cells in low serum medium containing 1 μM retinoic acid (RA) for 5 days induced formation of binucleated cells (Figure 1A, right panel). We quantified binucleation in differentiated H9c2 cells by DNA content using FACS. As shown in Figure 1B, RA-treated cells showed a dramatic increase in binucleated cells (73.8%) and a decrease in mononucleate DNA content (26.2%), compared with the controls. This system established a differentiated and binucleated H9c2 cell model induced by RA, as previously described [31]. Of interest, the RA treatment significantly increased SIRT1 protein and mRNA expression by about 9-fold in H9c2 cells (Figure 1C).

**Figure 2:**
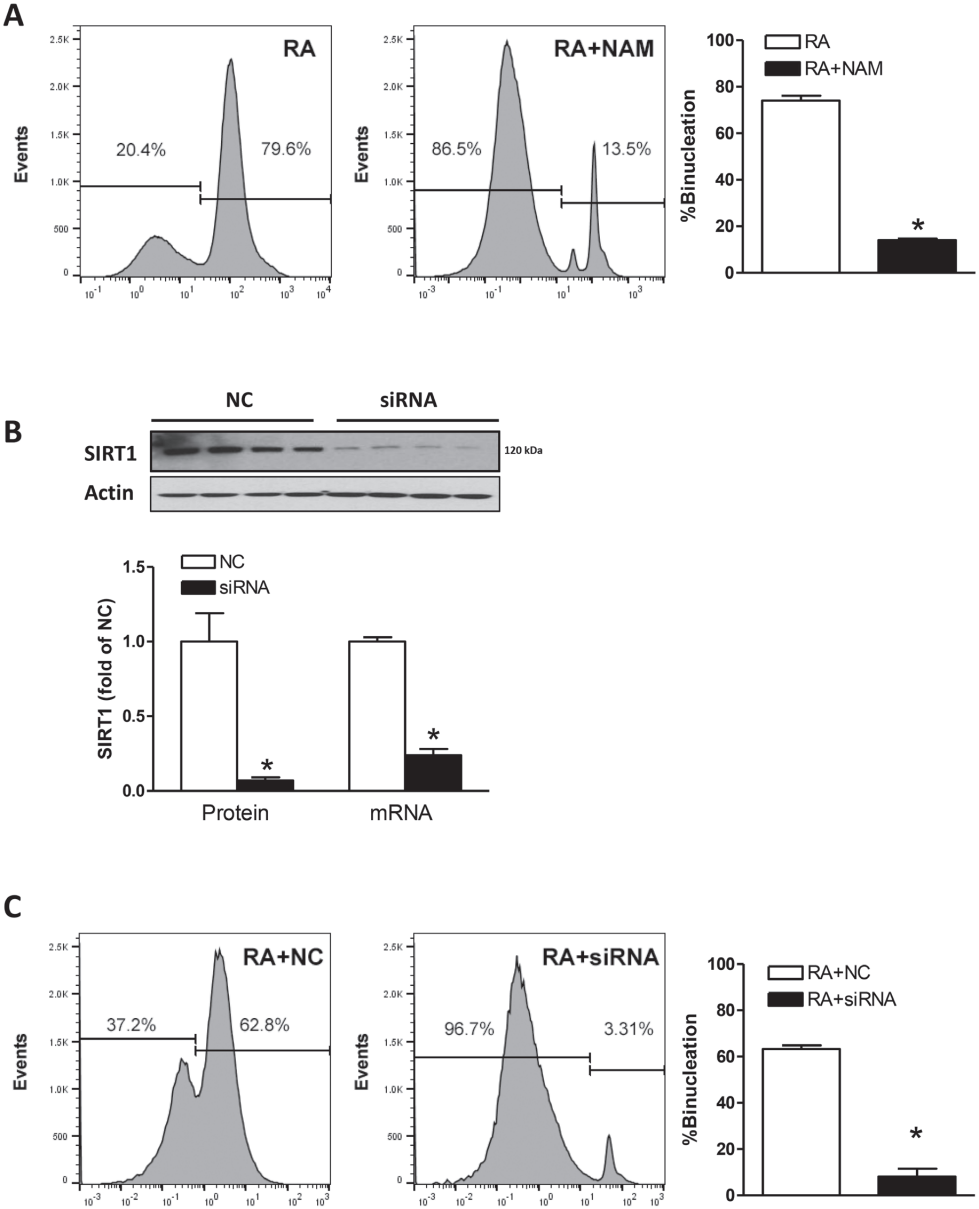
Effect of SIRT1 on binucleation (**A**) H9c2 cells were treated in the absence or presence of SIRT1 inhibitor nicotinamide (NAM; 10 mM) for 48 hours and binucleation was induced by retinoic acid for 5 days. Data are means ± SEM. *n* = 5 ^*^P < 0.05, RA + NAM versus RA. (**B**) H9c2 cell were transfected with 50 nM SIRT1 siRNAs or 50 nM siRNA negative controls (NC) for 48 hours. SIRT1 protein and mRNA abundance was determined with Western immunoblotting or RT-PCR. Data are the means ± SEM. *n* = 4 ^*^P < 0.05, siRNA *vs.* NC. (**C**) H9c2 cells were transfected with SIRT1 siRNAs or negative controls (NC) for 48 hours and binucleation was induced by retinoic acid for 5 days. Data are the means ± SEM. *n* = 5 ^*^P < 0.05, RA + siRNA *vs.* RA + NC.

To determine the causal role of SIRT1 in the binucleation, (a) SIRT1 activity was inhibited by nicotinamide (NAM), a potent inhibitor of SIRT1 activity [24], or (b) SIRT1 expression was suppressed using a siRNA approach. As shown in Figure 2A, NAM significantly inhibited RA-induced H9c2 cell binucleation. In addition, SIRT1 siRNA knocked down SIRT1 protein and mRNA abundance (Figure 2B) and drastically decreased RA-induced binucleation of H9c2 cells (Figure 2C), as compared with the negative control (NC). It is of note that RA-induced binucleation was slightly decreased in the presence of siRNA NC (Figure 2C), as compared with the absence of NC (Figure 2A).

### NAM reduced cardiomyocyte binucleation in neonatal rat hearts

**Figure 3:**
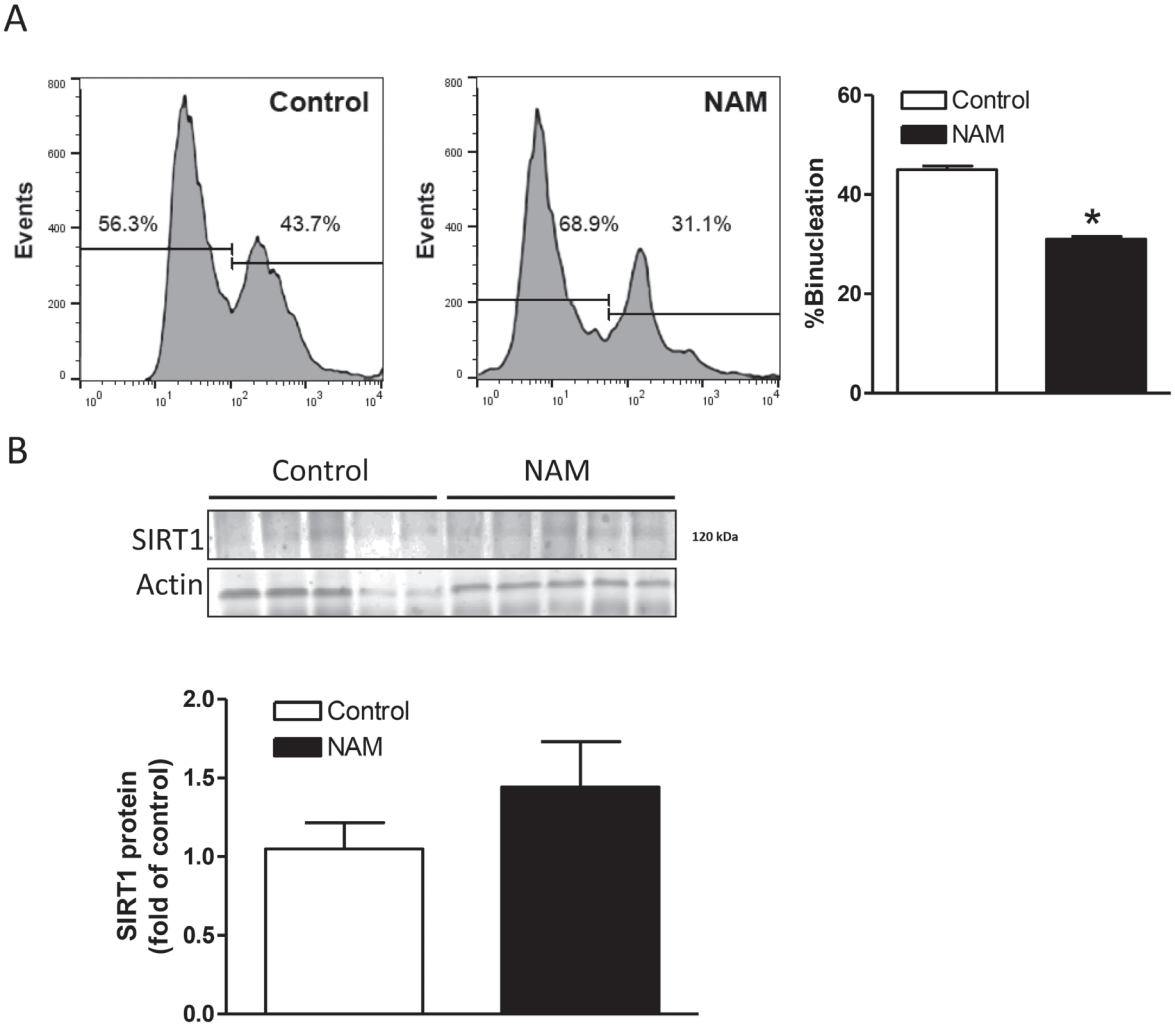
Effect of NAM treatment on SIRT1 expression and binucleation in the heart Nicotinamide (NAM, 500 mg/kg, i.p.) or saline (control) was administered to newborn rats daily from postnatal day 1 to 3. Hearts were isolated from postnatal day 7 pups. (**A**) Cardiomyocyte binucleation was determined with FACS analysis. (**B**) SIRT1 protein abundance was determined with Western immunoblotting. Data are means ± SEM. *n* = 5 ^*^P < 0.05, NAM versus control.

To determine the role of SIRT1 in cardiomyocyte binucleation *in vivo* in the developing heart, postnatal day 1 (P1) rat pups were treated with NAM or saline control once daily for 3 days. Cardiomyocytes were isolated from P7 pups and percentage of binucleation was determined by FACS analysis. In rodents, cardiomyocyte binucleation starts after birth and reaches around 40% in P7 pups [32]. The present finding of 43.7% binucleation in control P7 pups is consistent with our previous study [33] and that of others [32]. Compared with the saline control group, cardiomyocytes from NAM-treated pups demonstrated significantly less binucleation (31.1% in NAM-treated group *vs*. 43.7% in saline control) (Figure 3A). To demonstrate that NAM inhibited SIRT1 activity but not affected its expression levels, we measured SIRT1 protein abundance. As shown in Figure 3B, the NAM treatment did not have significant effect on SIRT1 protein abundance in the neonatal heart.

### Expression of SIRT1 and miR-133a in fetal and neonatal hearts

**Figure 4:**
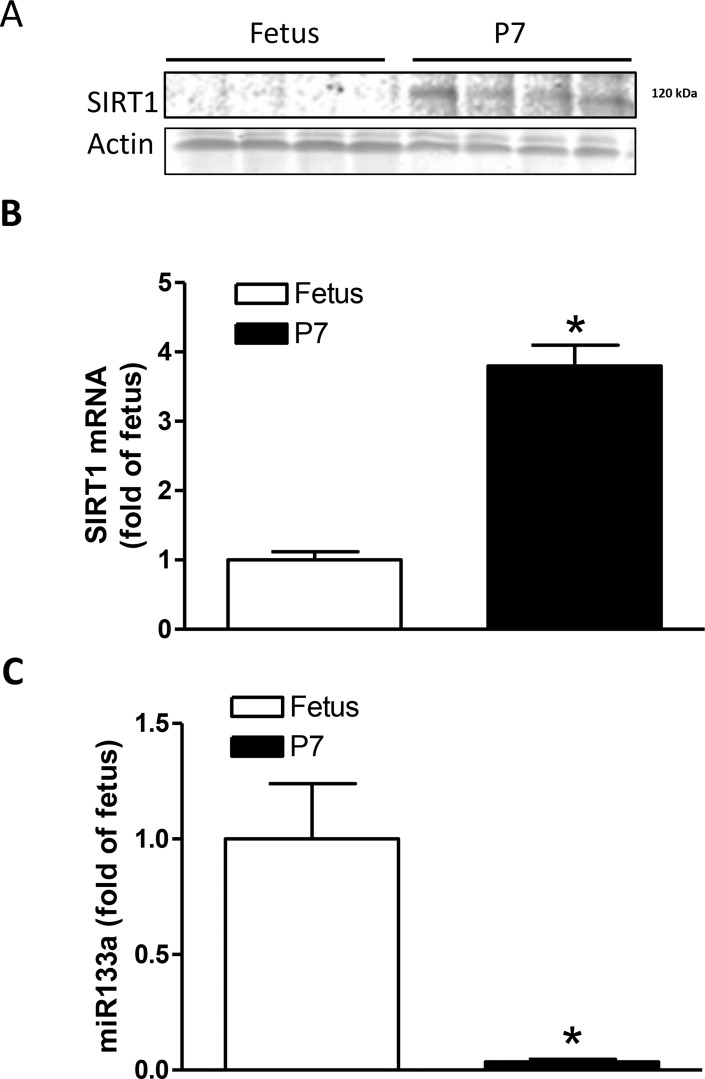
Expression of SIRT1 and miR133a in the developing heart Hearts were isolated from near-term (E21) fetuses and P7 pups and SIRT1 expression and miR133a abundance were determined. (**A**) SIRT1 protein abundance. (**B**) SIRT1 mRNA abundance. (**C**) miR133a abundance Data are means ± SEM. *n* = 4 ^*^P < 0.05, P7 pups versus fetuses.

Western blot analyses revealed that the expression of SIRT1 protein was extremely low in fetal hearts but significantly increased after birth in P7 neonatal hearts (Figure 4A). In addition, Figure 4B shows that SIRT1 mRNA abundance was significantly increased in P7 neonatal hearts. In contrast, the expression of miR-133a, a heart specific miRNA, was high in fetal hearts but significantly decreased in P7 hearts (Figure 4C), suggesting a possible role of miR-133a in the regulation of SIRT1 expression in the developing heart.

### MiR-133a directly targeted SIRT1 3′UTR

**Figure 5:**
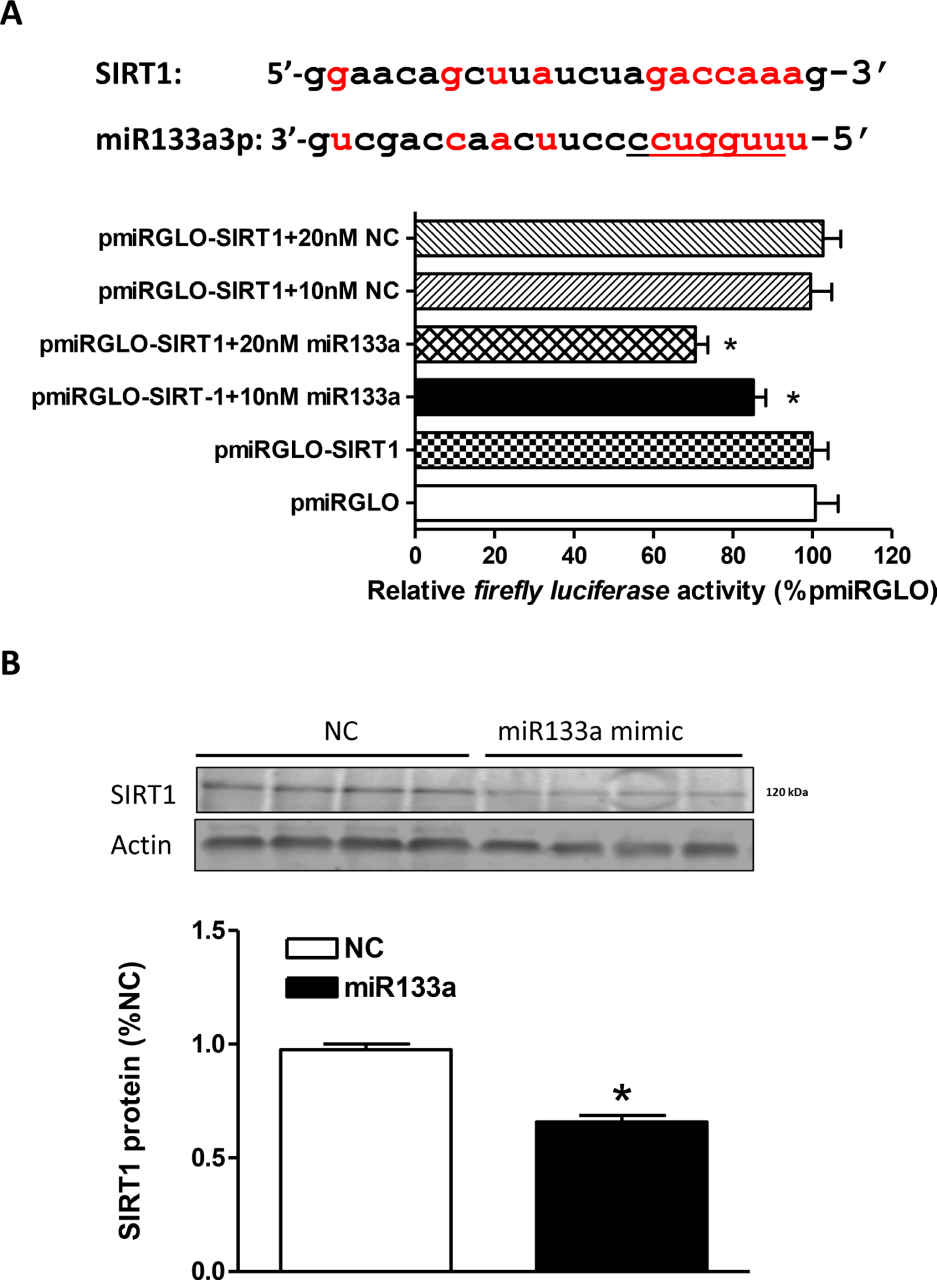
MiR133a binds to SIRT1 mRNA 3′UTR and inhibits SIRT1 protein expression (**A**) SIRT1 mRNA 3′UTR shows complementary binding sequences to the seed sequences of rat miR133a. H9c2 cells were transfected with pmiRGLO vector containing putative miR133a binding sites within the SIRT1 3′UTR (pmiRGLO-SIRT1) and co-transfected with miR133a mimic or negative control scramble for 48 hours. Luciferase activity was measured. Data are mean ± SEM. *n* = 6 ^*^P < 0.05, miR133a mimic versus pmiRGLO-SIRT1 alone. (**B**) SIRT1 protein abundance was measured in H9c2 cells transfected with miR133a mimic or negative control (NC) for 48 hour. Data are mean ± SEM. *n* = 4 ^*^P < 0.05, miR133a mimic versus negative control.

We identified a miR-133a-3p recognition motif in the rat SIRT1 transcript 3′UTR (Figure 5A). To determine if the interaction between miR-133a-3p mimic and SIRT1 mRNA is direct, we created a pmiRGLO-SIRT1 reporter construct and transfected H9c2 cells with the reporter construct. The reporter activity was measured in the absence or presence of miR-133a-3p mimic or the negative control. As shown in Figure 5A, the miR-133a-3p mimic produced a dose-dependent inhibition of reporter activity of the pmiRGLO-SIRT1 reporter construct, whereas the negative control had no effect. This was further demonstrated by the results showing that the miR-133a-3p mimic treatment significantly decreased SIRT1 protein abundance in H9c2 cells (Figure 5B).

### MiR-133a promoter was regulated by hypoxia response element (HRE)

**Figure 6:**
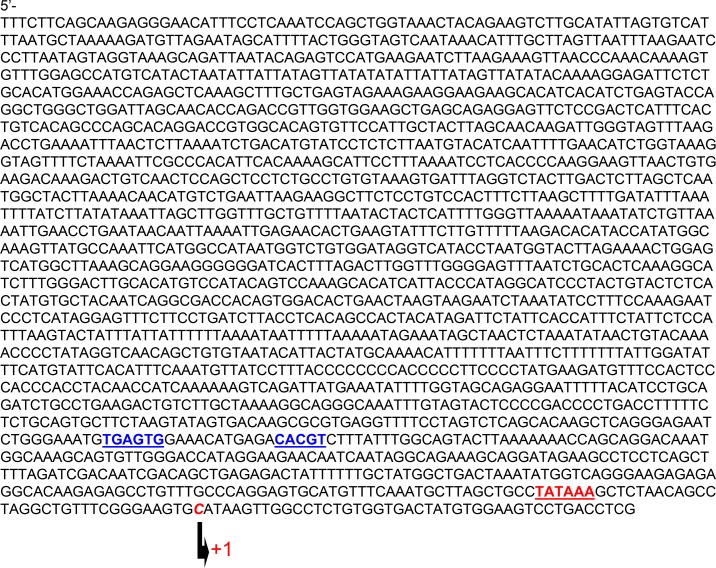
Sequences of rat miR1/133a gene bicistronic promoter The TATA BOX and the transcription start site are indicated in red font, while the hypoxia response elements (HRE) are indicated in blue font. The 5′end site of HRE is HBS motif and the 3′ end is HAS motif.

**Figure 7:**
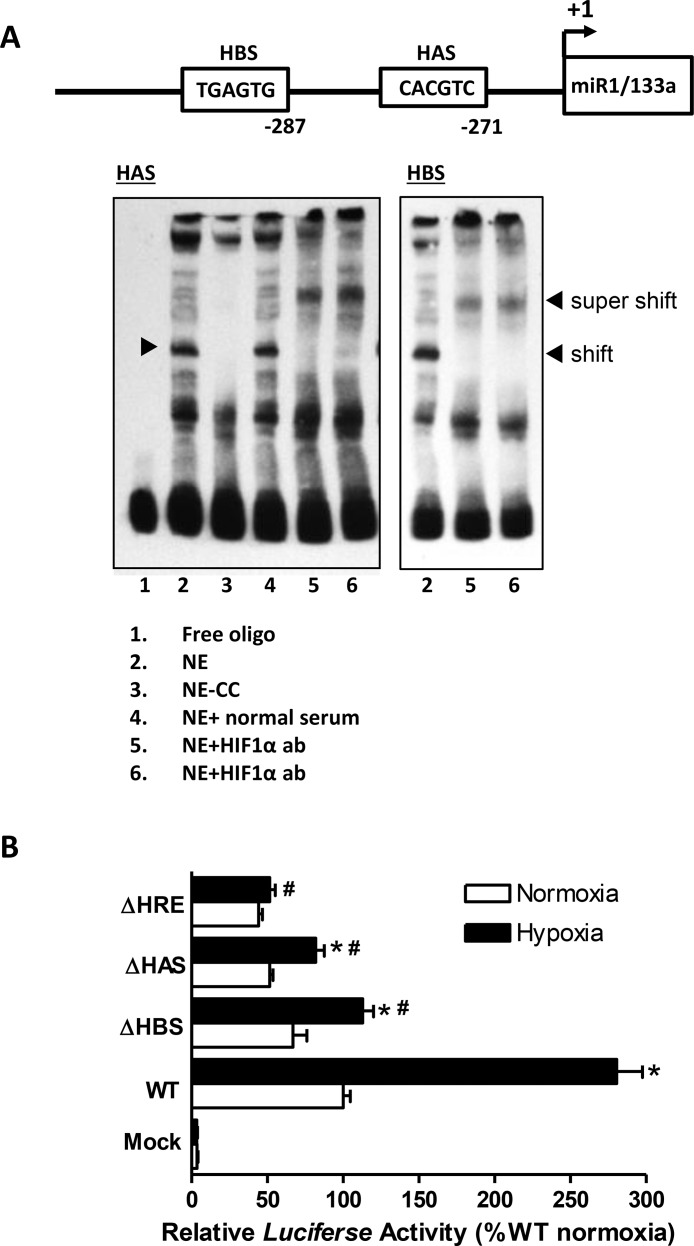
Identification and characterization of a unique HIF-1a binding site in the rat miR-1/133a bicistronic promoter (**A**) The schematic representation of rat miR1/133a bicistronic promoter region proximal to the transcription start site (+1) shows the presence of two HIF-1a binding sites: HAS and HBS. HIF-1a binding to the HAS and HBS sites in the miR-1/133a bicistronic promoter was demonstrated by EMSA and Super-shift assays. EMSA was performed with nuclear extracts (NE) from fetal hearts plus biotin labeled ds-oligo probes representing the HAS and HBS motifs in the absence or presence of HIF-1 a antibody (showing super-shift). CC: cold competition in the presence of 200-fold molar excess unlabeled ds-oligo probe. (**B**) Reporter gene constructs of wild-type (WT) miR-1/133a bicistronic promoter-pGL3 and three site-specific deletion constructs of HAS (ΔHAS), HBS (ΔHBS), or both (ΔHRE) were transiently co-transfected along with pRLSV40-*Luc* in H9c2 cells in normoxic (room air, ~20% O_2_) and hypoxic (1% O_2_) conditions. *Firefly* and *R. reniformis* luciferase activities in cell extracts were measured using a dual-luciferase reporter assay system. The promoter activities were calculated by normalizing the *firefly* luciferase to *R. reniformis* luciferase activity. Data are mean ± SEM. *n* = 6 ^*^p < 0.05, hypoxia versus normoxia; ^#^*p* < 0.05, deletions versus WT in hypoxia.

Given that fetal development takes place in a hypoxic environment and miR-133a expression is high in the fetal heart, we sought to examine whether miR-133a was regulated by hypoxia. We cloned the rat miR-1/133a bicistronic promoter (2043 base pair) from rat genomic DNA and identified a canonical hypoxia response element (HRE) with a bipartite HAS and a unique HBS motif and a classical TATAAA box [34] located at −37 bp upstream the transcription initiation site (Figure 6). To determine the binding of HIF-1α to miR-1/133a bicistronic promoter, we performed EMSA and super-shift assays. As shown in Figure 7A, both HAS and HBS oligo probes resulted in identical shift and super shift bands, confirming that HIF-1α interacted with both sites in the promoter. To further evaluate the functional significance of these binding sites in the regulation of miR-133 promoter activity, we performed reporter gene assays by site specific deletion of either HBS or HAS or both sites in the miR-133a promoter. Figure 7B shows that hypoxia significantly increased the promoter activity, which was inhibited by the deletion of either HBS or HAS or simultaneously both sites.

### Hypoxia upregulated miR-133a expression

**Figure 8:**
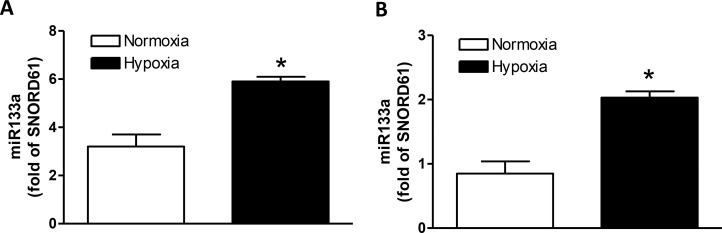
Effect of hypoxia on miR-133a expression in fetal hearts and cardiomyocytes (**A**) Hearts were isolated from E21 fetuses from pregnant rats treated with normoxia or hypoxia (10.5% O_2_) from day 15 to day 21 of gestation, and miR133a expression was measured by miScript miR real-time RT-qPCR. (**B**) Cardiomyocytes were isolated from fetal hearts and cultured myocytes were treated with normoxia or hypoxia (1% O_2_) for 24 hours. MiR133a expression was measured by miScript miR real-time RT-qPCR. Data are mean ± SEM. *n* = 5 ^*^p < 0.05, hypoxia versus normoxia.

To determine whether hypoxia indeed increased miR-133a in the heart *in vivo*, pregnant rats were treated with hypoxia (10.5% O_2_) from days 15 to 21 of gestation. Hearts were isolated from near-term (21-day) fetuses and miR-133a was measured. Compared with the control, the hypoxic treatment significantly increased miR-133a in the heart (Figure 8A). To study the direct effect of hypoxia on cardiomyocytes, hearts were isolated from day 21 fetal rats and cultured cardiomyocytes were treated with 1% O_2_ for 24 hours. As shown in Figure 8B, hypoxia significantly increased miR-133a in primary cardiomyocytes.

### Hypoxia downregulated SIRT1 expression

**Figure 9:**
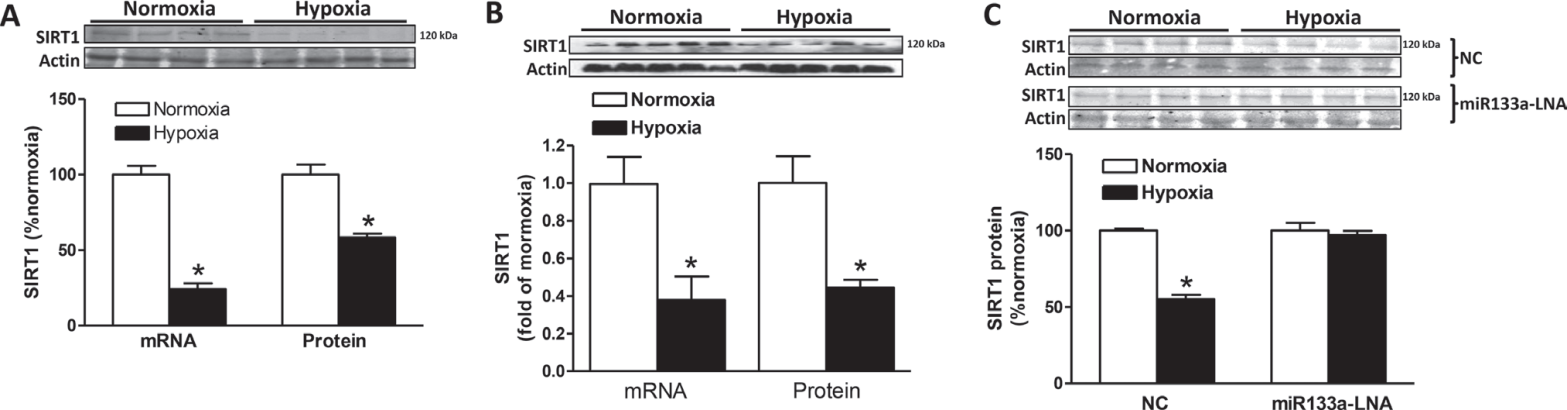
Effect of hypoxia on SIRT1 expression (**A**) SIRT1 mRNA and protein abundance was determined in H9c2 cells treated with normoxia (room air, ~20% O_2_) or hypoxia (1% O_2_) for 24 hours. Data are mean ± SEM. *n* = 4 ^*^P < 0.05, hypoxia versus normoxia. (**B**) Primary cardiomyocytes were isolated from E21 fetuses and treated with normoxia (room air, ~20% O_2_) or hypoxia (1% O_2_) for 24 hours. SIRT1 mRNA and protein abundance was determined. Data are mean ± SEM. *n* = 5 ^*^P < 0.05, hypoxia versus normoxia. (**C**) H9c2 cells were transfected with miR133a-LNA or the negative control (NC) for 48 hours followed by the treatment of normoxia or hypoxia (1% O_2_) for 24 hours. SIRT1 protein abundance was determined. Data are mean ± SEM. *n* = 4 ^*^P < 0.05, hypoxia versus normoxia.

As shown in Figure 9A, SIRT1 mRNA and protein abundance was significantly downregulated in H9c2 cells exposed to hypoxia (1% O_2_), as compared to the normoxic control. Similarly, the hypoxia treatment in primary cardiomyocytes isolated from day 21 fetal rats significantly decreased SIRT1 mRNA and protein abundance (Figure 9B). We then determined whether this hypoxia-induced downregulation of SIRT1 was mediated by miR-133a. As shown in Figure 9C, although the negative control had no effect on hypoxia-induced downregulation of SIRT1 protein abundance, the effect of hypoxia was blocked by the inhibition of miR-133a with the inhibitor miR-133a-LNA.

## DISCUSSION

In the present study, we provide evidence for a novel role of SIRT1 in the regulation of terminal differentiation and binucleation of cardiomyocytes in the heart development. Our results suggest that SIRT1 directly promotes binucleation of cardiomyocytes in the developing heart. When SIRT1 activity or expression was inhibited, cardiomyocyte binucleation was reduced. Furthermore, the study showed that hypoxia treatment resulted in a significant decrease in SIRT1 expression. This was consistent with the *in vivo* finding that SIRT1 expression was negligible in the fetal heart, and was upregulated in the heart of P7 neonatal pups. At the same time, we showed that mature miR-133a expression was high in fetal hearts, but significantly reduced in the hearts of day 7 pups. We further demonstrated that hypoxia stimulated miR-133a promoter activity and upregulated miR-133a expression in the heart, which in turn suppressed SIRT1 by binding to its transcript 3′UTR. Thus, the present study reveals a novel role of SIRT1, and its regulation by miR-133a, in cardiomyocyte terminal differentiation in the heart development.

It is widely recognized that hypoxia and HIF transcription factors play a significant role in the fetal development [25]. During the transition from fetal to postnatal life, the animal shifts from a hypoxic to normoxic environment. In the fetal heart, cardiomyocytes continue to proliferate until birth when cardiomyocytes begin to exit the cell cycle, become binucleated and terminally differentiate [3, 5]. The finding of extremely low expression of SIRT1 in the fetal heart and a significant increase of SIRT1 in the heart after birth shows the similar pattern with the transition of cardiomyocytes from mononucleated to binucleated and suggests a role of SIRT1 in the heart development. Of importance, the causal role of SIRT1 was demonstrated in the present study by the findings that inhibition of SIRT1 retarded the binucleation process in myocytes as well as *in vivo* in neonatal hearts. This was further demonstrated by the finding that knockdown of the SIRT1 gene in H9c2 cells significantly decreased the RA-induced binucleation. Whereas the present studies of inhibition of SIRT1 and knockdown of the SIRT1 gene provide clear evidence of a role of SIRT1 in cardiomyocyte binucleation, future studies may consider to over-express the SIRT1 gene in cardiomyocytes, which may provide additional information. In rodents, cardiomyocyte maturation and terminal differentiation occur over the first two weeks of postnatal life [1, 5, 6] and have been shown to increase in binucleation in a stepwise manner over this time period [1, 32]. Our finding that binucleated cardiomyocytes were around 44% in P7 neonatal hearts is consistent with the previous findings [32, 33].

The terminal differentiation of cardiomyocytes is characterized by an exit from the cell cycle leading to a loss of proliferation. The vast majority of adult cardiomyocytes are shown to be arrested in the G0/G1 phase [32, 35]. The physiological importance and mechanisms by which binucleation occurs are still poorly understood. Possible explanations suggest that binucleation allows cardiomyocytes to (a) better cope with stress [36] and/or (b) to meet their high metabolic demand by producing twice the amount of RNA and protein [5]. SIRT1 has been shown to promote survival as a defensive mechanism to prevent stress-induced cell death in myoblasts [37], and is involved in several processes including: cardiovascular disease, neurodegeneration, inflammation, cancer, and regulation of metabolism [38–40]. It is thought to modulate the process of aging by increasing resistance to stress [9, 41] and protecting against oxidative stress [9, 13]. These are consistent with its role in promoting cardiomyocyte binucleation, i.e. increasing the heart's ability against energy stress and oxidative stress after birth. However, the role of SIRT1 as protective against oxidative stress as well as its role in the conservation of energy demand in the developing heart require further investigation. During the development, mice deficient in SIRT1 have abnormalities of the heart as well as other organs and rarely survive after birth [42, 43].

The mechanisms underlying SIRT1-induced cardiomyocyte binucleation remain to be determined. SIRT1 is a Class III histone deacetylase. Histone deacetylation plays an important role in the stable silencing of cell cycle genes specifically involved in the progression of G2/M transition and cytokinesis that occurs in cardiomyocyte terminal differentiation [44]. In addition to histone deacetylation, SIRT1 also functions as a non-histone protein deacetylase and has been shown to regulate several transcription factors *via* deacetylation, including FOXO [41, 45, 46] and HIF1α [23]. SIRT1 has been shown to deacetylate FOXO3a and thereby alter its activity [12, 41]. Interestingly, SIRT1 regulates FOXO3a's activity by (a) increasing its ability to induce cell cycle arrest and (b) inhibiting its ability to induce apoptosis [41], thus providing protection against oxidative stress [13, 41]. SIRT1 can also deacetylate FOXO1 in cardiomyocytes, repress its activity, and thus inhibit apoptosis [45, 46]. Our recent study demonstrated that glucocorticoids promoted cardiomyocyte terminal differentiation *via* the repression of cyclin D2 [47]. It has been shown that activation of SIRT1 by resveratrol, a known activator of SIRT1 [48], leads to decreased expression of various cyclins in several cancer cell lines [49]. Taken together, these studies may suggest potential mechanisms through which SIRT1 regulates the terminal differentiation process associated with binucleation.

The finding of an inverse relationship between SIRT1 and miR-133a expression patterns in the developing heart is of interest and suggests a regulation of SIRT1 by miR-133a during the heart development. Indeed, we demonstrated that SIRT1 was a direct target of miR-133a that downregulated SIRT1 expression. Similarly, it was reported that miR-133 targeted SIRT1 in glioma cells, leading to inhibition of cell proliferation [50]. In the present study, we found that miR-133a was significantly upregulated in both fetal hearts and cardiomyocytes exposed to hypoxia, which coincides with the fact that the *in utero* environment is naturally hypoxic. To our knowledge, we are the first to identify a canonical Hypoxia Response Element (HRE) at the miR-1/133a bicistronic promoter and its function in the regulation of the promoter activity in response to hypoxia. This bicistronic promoter drives the transcription of two heart specific microRNAs, namely miR-1 and miR-133a. MiRNA-133a has been shown to play an important role in cardiac development [29]. The present study suggests a new mechanism by which miR-133a regulates cardiomyocyte terminal differentiation *via* suppressing SIRT1 expression. In addition, a previous study showed that miR-133a regulated cardiac cell proliferation via inhibiting cyclin D2 [51], which may also play a role in cardiomyocyte differentiation. Of interest, HIF-1α is inhibited by SIRT1 deacetylation [23] – this may suggest a possible positive feedback mechanism through which the development-dependent increase in binucleation occurs at the critical window around birth. It is possible that the decrease in HIF-1α following birth may promote SIRT1 expression in the heart, which elicits a positive feedback to further decrease HIF-1α function to ensure binucleation over this critical period of heart development after birth. Nonetheless, this possibility needs to be further determined as the relatively low level of oxygen *in utero* is considered physiologically “normoxic” to the fetus.

Overall, the present study demonstrates for the first time an axis of hypoxia-miR-133a-SIRT1 in the regulation of cardiomyocyte terminal differentiation during the heart development. Indeed, the transition to an oxygen-rich environment after birth is of critical importance in inducing cardiac muscle cells to stop proliferating in mammals [52]. The present study provides evidence of a novel mechanism of miR-133a/SIRT1 through which changing oxygen levels affect the timing of cell-cycle withdrawal in the developing heart. This may not exclude the possibility for SIRT1 responses directly to hypoxia. It should be noted that the heart development is somewhat different between rodents and humans, and thus the findings in rodents may not be able to directly apply to humans. Nonetheless, revealing the mechanisms involved in cardiomyocyte differentiation should provide new insights into potential therapeutic strategies in the regeneration of heart muscle after injury or disease in adulthood. Indeed, it has been demonstrated recently that hypoxia elicits heart regeneration in adult mice by inducing the re-entry of cell cycle and the proliferation of pre-existing cardiomyocytes [53].

## MATERIALS AND METHODS

### H9C2 cell culture differentiation and hypoxia treatment

H9c2 cells were plated as previously described [31]. Cells were cultured for a day in high serum (10%) to allow for attachment. The terminal differentiation and binucleation of H9c2 cells were induced by retinoic acid, as previously described [31, 54]. Serum in the media was reduced to 1% to initiate H9c2 cell differentiation, followed by the addition of 1μM all-trans-retinoic acid [31, 54]. Cells were treated in the dark for 5 days. For the hypoxia treatment, cells were placed into an anaerobic chamber and exposed to 1% O_2_ for 24 hours. Normoxic control was under room air (~20% O_2_).

### Experimental animals and drug treatment

All-trans-retinoic acid was purchased from Sigma-Aldrich. Pregnant Sprague-Dawley rats were purchased from Charles River Laboratories (Portage, MI). Animals were allowed to give birth and pups were randomly divided into the saline control and nicotinamide (NAM)-treated groups. Pups from different litters were given intraperitoneal injections with NAM (500 mg/kg, *n* = 5) or saline (*n* = 5) [55, 56] once daily for the first 3 postnatal days. Pups were then anesthetized using isoflurane and hearts were removed for isolation of cardiomyocytes on postnatal day 7 (P7) to determine cardiomyocyte binucleation by flow cytometry. For the *in vivo* hypoxia treatment, pregnant rats were treated with normoxia in room air or hypoxia (10.5% O_2_) from days 15 to 21 of gestation. Hearts were isolated from near-term (21 day) fetuses. For Western blot analysis or miR133a measurement, tissue homogenates were prepared from the whole hearts isolated from 21 day fetuses and P7 pups. Total of 10 litters and 10 pregnant dams were used in the studies. All procedures and protocols were approved by the Institutional Animal Care and Use Committee of Loma Linda University and followed the guidelines by the National Institutes of Health Guide for the Care and Use of Laboratory Animals.

### Primary cardiomyocyte isolation and culture

Cardiomyocytes were isolated from hearts by enzymatic digestion, as previously described [47, 57–59]. Cells were cultured and plated on 6-well plates, some wells contained poly-L-lysine (Sigma, St. Louis, MO)-coated coverslips.

### siRNA transfection

To silence the SIRT1 gene, siRNAs designed to specifically knock down rat *SIRT1* or siRNA negative controls were purchased from Dharmacon (Lafayette, CO). Cell transfection was performed with HiPerfect transfection reagent (Qiagen, Valencia, CA) following the manufacturer's recommendations, as described previously [60]. Briefly, the mixture of target or negative siRNAs, Briefly, the mixture of target or negative siRNAs, HiPerfect transfection reagent, and Minimum Essential Media I (Thermo Fisher, Waltham, MA) was prepared and incubated for 10 minutes at room temperature. H9c2 cells were transfected with 50 nM SIRT1 siRNAs or 50 nM siRNA negative controls for 48 hours. Cells were subjected to further analysis 48 hours after transfection.

### Real-time RT-PCR

RNA was extracted from rat hearts, isolated primary cardiomyocytes and H9C2 cells. SIRT1 mRNA abundance was determined by real-time RT-PCR using an Icycler Thermal cycler (Bio-Rad, Hercules, CA), as described previously [61]. Real-time RT-PCR was performed in a final volume of 25 μL. RT-PCR primers for rat SIRT1 are, *forward*: 5′-gataccttggagcaggttgc, and *reverse*: 5′-cacctaggacaccgaggaac. RT-PCR primers for the internal control ribosomal RNA 18S are, *forward*: 5′-gcaattattccccatgaacg, *reverse*: 5′-ggcctcactaaaccatccaa. Polymerase chain reaction was performed in triplicate and threshold cycle numbers were averaged. MiR-133a was measured using the miScript SYBR Green PCR kit (Qiagen). SNORD61 was used as an internal control.

### Flow cytometry

Cardiomyocyte binucleation was analyzed by flow cytometry, as previously described [32]. Cells were fixed and incubated with propidium iodide (PI) and rat anti-troponin T, FITC conjugated. Rat spleen cells were used as a mononucleate cell control. Flow cytometry analysis was performed using a MACSQuant analyzer (Miltenyi Biotec Inc., Auburn, CA, USA) and FlowJo analysis software (FlowJo, Ashland, OR, USA).

### Western immunoblotting

Western blots were performed as previously described [61]. Briefly, 30–50 μg proteins were separated in 10% polyacrylamide gel, transferred onto nitrocellulose membranes and probed with a primary antibody against SIRT1 (1:1000; Cell Signaling, Danvers, MA). After washing, membranes were incubated with the secondary horseradish peroxidase (HRP)-conjugated goat anti-mouse antibody (1:2,000). Signals were detected on an Odyssey Infrared Imager (LI-COR Bioscience, USA). The results were analyzed with Image J analysis software, and were normalized to β-actin.

### Confocal microscopy

Cells were fixed, permeabilized, stained with Hoechst and examined with a Zeiss LSM710 confocal microscope.

### SIRT1 3′UTR cloning and luciferase reporter gene assays

We identified a rat miR-133a recognition motif in the rat sirtuin 1 (SIRT1) transcript 3′UTR. A 412-bp segment of 3′UTR of rat SIRT1 mRNA containing the miR-133a binding sequence was PCR amplified as previously described [61]. The segment was inserted into pmirGLO luciferase vector (Promega) to create a pmirGLO-SIRT1 reporter construct, which was transfected into H9c2 cells. The *Firefly* and *Renilla reniformis luciferase* activities were measured using a dual-luciferase reporter assay kit (Promega). The *Firefly luciferase* activity was normalized to *Renilla reniformis luciferase* activity and expressed as relative to control pmirGLO-SIRT1 activity, as previously reported [61].

### Analysis of rat miR-1/133a bicistronic promoter

MiR-1 and miR-133a are coded by a bicistronic promoter [29]. The sequence information of this bicistronic promoter was obtained from rat chromosome 18 available at Genbank, (https://www.ncbi.nlm.nih.gov/gene/100314030). A 2043 base pair (bp) rat miR-1/miR-133a bicistronic promoter sequence was amplified by preparative PCR starting from 100 ng rat genomic DNA using forward and reverse primers, and was cloned in pCR 4-TOPO vector (Invitrogen). For the purpose of reporter gene assay, the (5′)-NheI-2043 bp amplicon-XhoI -(3′) was cloned into the *luciferase* reporter plasmid pGL3 basic vector (Promega) to generate the full-length promoter-reporter plasmid containing −1099 bp to +44 bp relative to the transcriptional start site. Bioinformatics Promoter analyses (Genomatix) identified a bipartite (HBS and HAS) canonical hypoxia response element (HRE) at −293 bp and a TATA box at −37 bp upstream the transcription start site. Reporter gene assay was performed using H9c2 cells, as described previously [62]. Cells were transiently co-transfected with 1μg of promoter-reporter construct along with 0.05 μg of internal normalizer control pRL-SV40 Luc vector (Promega). After 48 hours, firefly and *Renilla reniformis* luciferase activities in cell extracts were measured with a luminometer (Berthold Technologies) using a dual-luciferase reporter assay system (Promega). Promoter activities of WT and deletion constructs were then calculated by normalizing the *firefly luciferase* activities (pGL3 constructs) to *Renilla reniformis luciferase* activity of pRL-SV40 Luc vector as described previously [62].

### Electrophoretic mobility shift assay (EMSA)

Nuclear extract (NE) was prepared from fetal rat hearts using NXTRACT CelLytic Nuclear Extraction Kit (Sigma). The oligonucleotide probes containing HBS (sense: 5′-ctgggaaatgtgagtggaaacatgag; anti-sense: 5′-ctcatgtttccactcacatttcccag) and HAS (sense: 5′-gaaacatgagacacgtctttatttggca; anti-sense: 5′-tgccaaataaagacgtgtctcatgtttc) motifs in the miR-133a promoter were biotin-labeled and then subjected to EMSA using LightShift Chemiluminescent EMSA Kit (Pierce Biotechnology). For cold competition (CC), 200-fold molar excess of unlabeled homologous oligonucleotides were added to EMSA binding reactions. As described earlier [63], for super-shift assays, 1 μl of normal rabbit serum or HIF-1α antiserum (Active Motif) was pre-incubated with NE in the 1x binding buffer for 2 hours at 4°C. This was followed by addition of biotinylated probe and poly (dI-dC). The mixture was separated by electrophoresis and transferred to a Biodyne nylon membrane (Pierce). Membranes were blocked and the shifted or super-shifted bands were visualized using the reagents provided in the LightShift kit (Pierce) following Pierce protocol and recorded on an x-ray film.

### Statistical analysis

Data are expressed as mean ± SEM. For the normalization of data to controls or negative controls, the average was first obtained for the control group, followed by normalization of all data to the average. Statistical analysis (*p* < 0.05) was determined by two-way analysis of variance (ANOVA) followed by Newman-Keuls post hoc test or Student's *t* test, where appropriate.

## Abbreviations

SIRT1: silent information regulator of transcription
RA: retinoic acid
P7: postnatal day 7
HRE: hypoxia response element
miR-133a: microRNA-133a
3′UTR: 3′-untranslated region
NAD^+^: nicotinamide adenine dinucleotide
NAM: nicotinamide
HIF: hypoxia inducible factor
FACS: fluorescence-activated cell sorting
siRNA: small interfering RNA
EMSA: electrophoretic mobility shift assay
HAS: HIF-1 ancillary sequence
HBS: HIF-1 binding site
HIF-1α: hypoxia-inducible factor 1-alpha
LNA: locked nucleic acid
FOXO: forkhead box protein
